# An ADaptivE PrenaTal (ADEPT) intervention to increase childhood vaccinations: Protocol for a cluster randomized trial and nested mixed methods evaluation

**DOI:** 10.1371/journal.pone.0313742

**Published:** 2024-11-21

**Authors:** Lavanya Vasudevan, Rachael M. Porter, Ilse Campos, Elizabeth L. Turner, Sandra S. Stinnett, Leah L. Zullig, Emmanuel B. Walter, Geeta K. Swamy, Robert A Bednarczyk, Walter A. Orenstein, Beverly Gray

**Affiliations:** 1 Hubert Department of Global Health, Emory University, Atlanta, Georgia, United States of America; 2 Duke Global Health Institute, Durham, North Carolina, United States of America; 3 Department of Biostatistics and Bioinformatics, Duke University, Durham, North Carolina, United States of America; 4 Department of Population Health Sciences, Duke University, Durham, North Carolina, United States of America; 5 Center of Innovation to Accelerate Discovery and Practice Transformation (ADAPT), Durham Veterans Affairs Health Care System, Durham, North Carolina, United States of America; 6 Department of Pediatrics, Duke University School of Medicine, Durham, North Carolina, United States of America; 7 Duke Human Vaccine Institute, Durham, North Carolina, United States of America; 8 Department of Obstetrics and Gynecology, Duke University, Durham, North Carolina, United States of America; 9 Department of Medicine, Emory University, Atlanta, Georgia, United States of America; PLOS: Public Library of Science, UNITED KINGDOM OF GREAT BRITAIN AND NORTHERN IRELAND

## Abstract

**Background:**

There is limited evidence to assess if interventions implemented during pregnancy proactively mitigate parental vaccine hesitancy and promote timely vaccination among children after birth. This study protocol describes the evaluation of an ADaptivE PrenaTal (ADEPT) intervention to increase childhood vaccinations that is implemented with first-time pregnant individuals (PIs).

**Methods:**

Within the framework of a type 1 effectiveness-implementation hybrid study design, a cluster-randomized trial (CRT) will determine the effectiveness of ADEPT at increasing childhood vaccinations, and a nested explanatory mixed methods (NMM) study will assess changes in parental vaccine hesitancy. Study practices will be randomized to deliver ADEPT in addition to standard of care or standard of care alone. Providers at intervention sites will participate in a 4-part training program on childhood vaccines and effective communication. During a routine prenatal visit, providers will discuss vaccines recommended for the PI during pregnancy and for the child after birth, following which PIs will be screened for vaccination intention. Vaccine-hesitant PIs will be offered adaptive components of the intervention, which include an educational website and phone call with a vaccine navigator to discuss concerns. They will also be offered enrollment into the NMM study, where their vaccination intention will be assessed post-intervention. After PIs give birth, their child’s vaccination outcomes at 2 months will be extracted from the state immunization registry. The primary study outcome is the difference in timely childhood vaccination at 2 months between the intervention and control arms. The secondary outcome is reduction in vaccine hesitancy assessed among PIs in the NMM study as the pre-post intervention change in vaccination intention.

**Discussion:**

The study findings are expected to contribute evidence on the effectiveness of prenatal interventions to proactively mitigate parental vaccine hesitancy and promote timely vaccinations after the child’s birth.

**Trial registration:**

The study protocol is registered in ClinicalTrials.gov (NCT05795855).

## Background

Among children born in the United States (US) from 2020–2022, an estimated 30% were not up-to-date with recommended vaccines by 24 months of age [1]. Prior publications have documented varied patterns of adherence to the recommended vaccination schedule in the US, including delayed and selective vaccination, and refusal of all vaccines [2–4]. Delayed vaccinations increase the period that children remain at risk for preventable infections such as measles and polio, and efficacy of vaccine protection is reduced when all recommended vaccine doses are not received [2]. Findings from a 2022–23 survey by the American Academy of Pediatrics suggest an increase in requests to deviate from the recommended vaccine schedule, with a majority (>90%) of surveyed pediatricians reporting such requests from at least one family in their practice in the 12 months preceding the survey [5].

Parents express a variety of reasons for delayed or refused vaccinations among children, including concerns about vaccine safety, complacency toward vaccine-preventable diseases, and concerns about the number of vaccines a child gets in a single visit [6, 7]. Concerns about the number of vaccines in particular have been compounded by the introduction of novel vaccines and therapeutics in the US childhood vaccination schedule, including the mRNA-based COVID-19 vaccine annually starting at 6 months of age, and a monoclonal antibody injection for infants <8 months old to protect against Respiratory Syncytial Virus (RSV). In a Pew Research survey, half of parents of children aged 0–4 years were somewhat concerned about the necessity of all recommended vaccines [8]. Since recommended vaccines protect children from serious, and sometimes deadly, infections, responding to parental concerns is critical for supporting timely vaccinations and reducing preventable childhood morbidity and mortality.

Pregnancy is an under-utilized time for parental vaccine education. In a 2022 survey focused on pregnant individuals in prenatal practices associated with one academic medical center in North Carolina, we found only 67% with an intention to accept all vaccines (not inclusive of the COVID-19 vaccine) for their child after birth, and only 35% with an intention to accept COVID-19 vaccines for their child, when eligible [9]. Existing interventions aimed at reducing parental vaccine concerns focus on the pediatric primary care setting, in the period after the child is born [10–12]. Since vaccines are routinely recommended during the prenatal setting for the pregnant individual, there is an opportunity to expand vaccine conversations during pregnancy to address childhood vaccinations [13–15]. Routine prenatal care includes multiple interactions with the healthcare system that present opportunities for timely intervention on childhood vaccination. However, there are no guidelines in the current standard of care for the inclusion of childhood vaccine conversations during pregnancy [14–16]. In addition, prenatal care providers are rarely prioritized for formal education about childhood vaccines [17]. In a survey conducted in April 2022, prenatal providers identified time constraints during appointments and training gaps on childhood vaccines as top barriers to implementation of a childhood vaccination intervention [18]. Evidence on the effectiveness of prenatal interventions that incorporate provider training and account for constraints on provider time is needed to transform clinical practice guidelines for inclusion of childhood vaccine conversations.

This protocol describes plans to implement and evaluate an ADaptivE PrenaTal (ADEPT) intervention to increase childhood vaccinations. ADEPT is a multi-component adaptive intervention designed to support first-time pregnant individuals (PIs) in their decisions around vaccinations in pregnancy and for the child after birth. This protocol describes key features of the ADEPT intervention and plans for its evaluation in a type 1 effectiveness-implementation hybrid study.

## Methods

**Fig 1:**
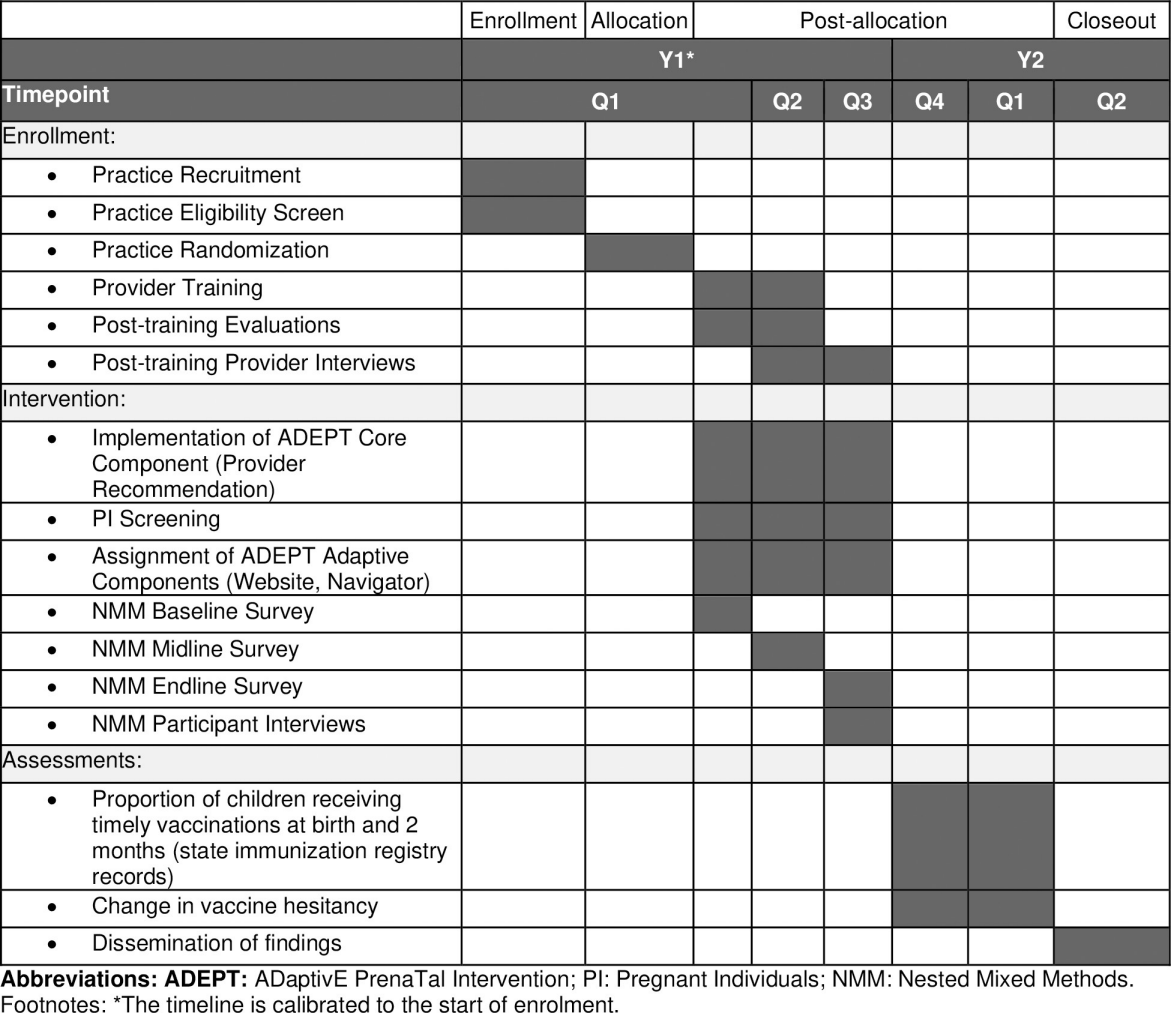
SPIRIT schedule of enrollment, interventions, assessments for the ADEPT study. Activities related to practices, providers, and pregnant individuals.

The evaluation of the ADEPT intervention is described in accordance with the SPIRIT schedule of enrollment, interventions, and assessments (**Fig 1** and **S1 File**) [19]. Prospective recruitment of study practices started on April 27, 2024, and is expected to be completed by January 31, 2025.

### Description of the intervention

**Fig 2:**
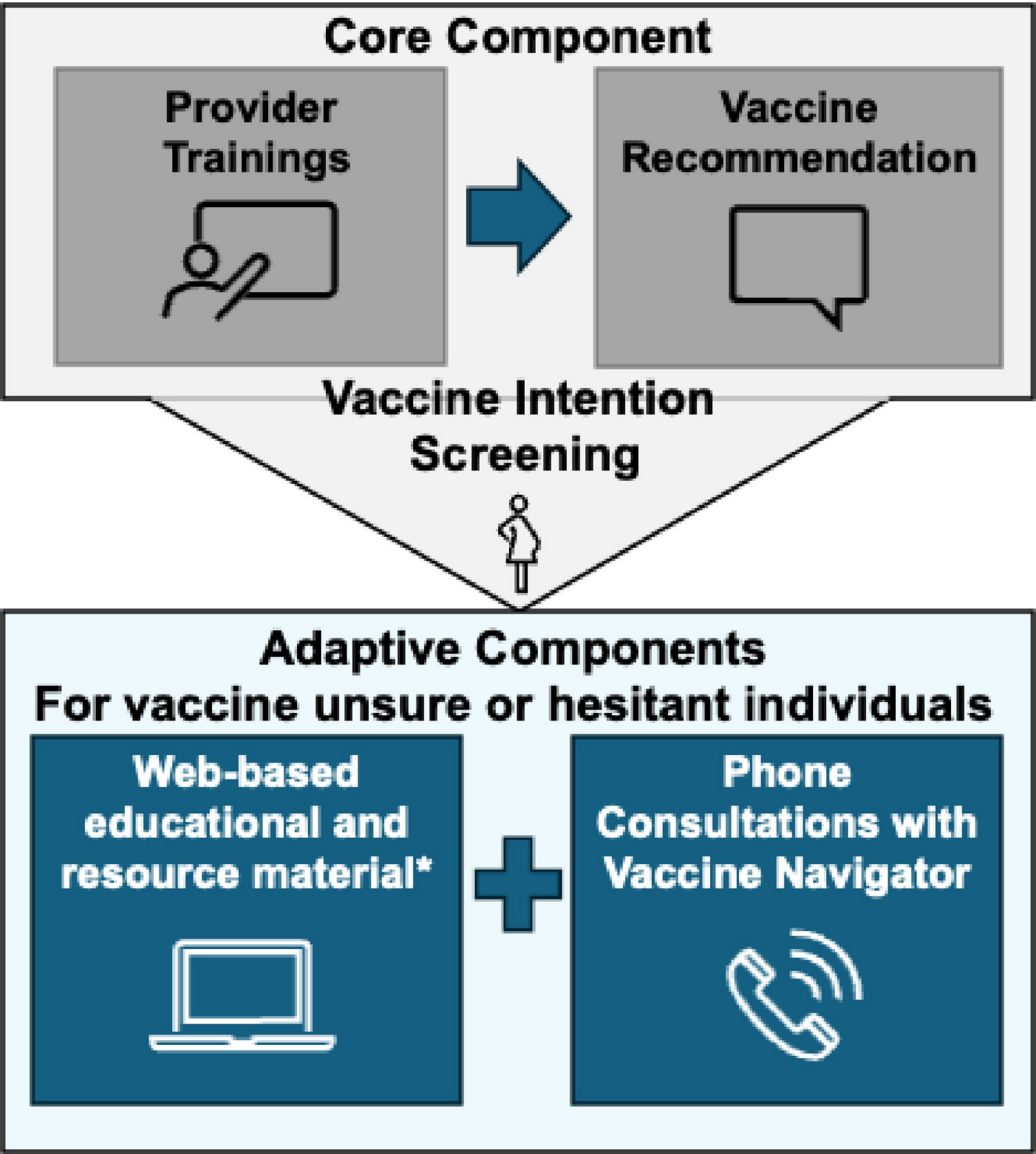
ADEPT core and adaptive components.

The ADEPT intervention components are described in accordance with the template for intervention description and replication (TiDieR) checklist (**S2 File**) [20]. The core and adaptive components of ADEPT are shown in **Fig 2**. The core component of ADEPT is a prenatal provider recommendation in favor of maternal and childhood vaccinations. Adaptive components include a multimedia educational website and phone consultations with a vaccine navigator. These components are described below.

#### Core component

The core component of ADEPT is a vaccine recommendation by prenatal providers to first-time PIs at the end of the second trimester (26–32 weeks), encouraging vaccinations during pregnancy and for their child after birth. Prior to intervention implementation, providers at intervention sites will complete four training sessions on the following topics: overview of the routine vaccination program in the US (including COVID-19 vaccine and RSV immunization recommendations), vaccine clinical trials and safety, and strategies for effective communication (2 sessions). During active implementation, prenatal providers will make recommendations encouraging timely maternal and childhood vaccinations.

#### Adaptive components

The ADEPT intervention consists of two adaptive components: a multimedia vaccine education website and phone consultations with a vaccine navigator. The assignment of the adaptive component is determined based on a screening questionnaire that will assess vaccination intention, that is administered after the core component is delivered to the PI. PIs who have a positive vaccination intention will not receive any adaptive intervention components. PIs who are in favor of vaccinations but exhibit some degree of hesitancy (e.g., plan to delay some vaccines) will be offered access to the multimedia vaccine education website. PIs with negative intention to vaccinate or those unsure of their decision will be offered access to the multimedia vaccine education website, as well as a phone consultation with a vaccine navigator.

- Multimedia vaccine education website: An educational website was co-developed with parents. The website includes the following sections: About Us, About Vaccines, Vaccine Preventable Diseases, About the Schedule, Vaccination Schedule, Vaccines During Pregnancy, Visit Detail, What Doctors Say, Research with Confidence, Vaccines by Numbers, and More Resources. The ‘More Resources’ tab includes links to other credible websites such as cdc.gov and the American Academy of Pediatrics’ Healthy Children webpage for those interested in learning more. PIs without sufficient access to the internet or an internet-enabled device, may request access to a handout with the website content. Such individuals will also be reassigned automatically to receive a phone consultation with a vaccine navigator.
- Vaccine Navigator: The navigators are members of the study team and will connect with eligible PIs via phone. The vaccine navigators will participate in the provider trainings described with the core component of the intervention. The purpose of the navigator is to respond to questions or concerns not addressed by the website or during provider recommendation.

### Study design overview

ADEPT will be evaluated using a type 1 effectiveness-implementation hybrid study design that includes a cluster randomized trial to assess effectiveness outcomes and a nested mixed method study to assess changes in parental vaccine hesitancy following exposure to the intervention [21]. The methods below are described in accordance with the Consort 2010 statement: extension to cluster randomized trials (**S3 File**) [22].

### Evaluation objectives

The primary objective of the evaluation is to determine the effectiveness of ADEPT at increasing timely childhood vaccinations compared to standard of care.

#### Cluster-Randomized Trial (CRT)

**Fig 3:**
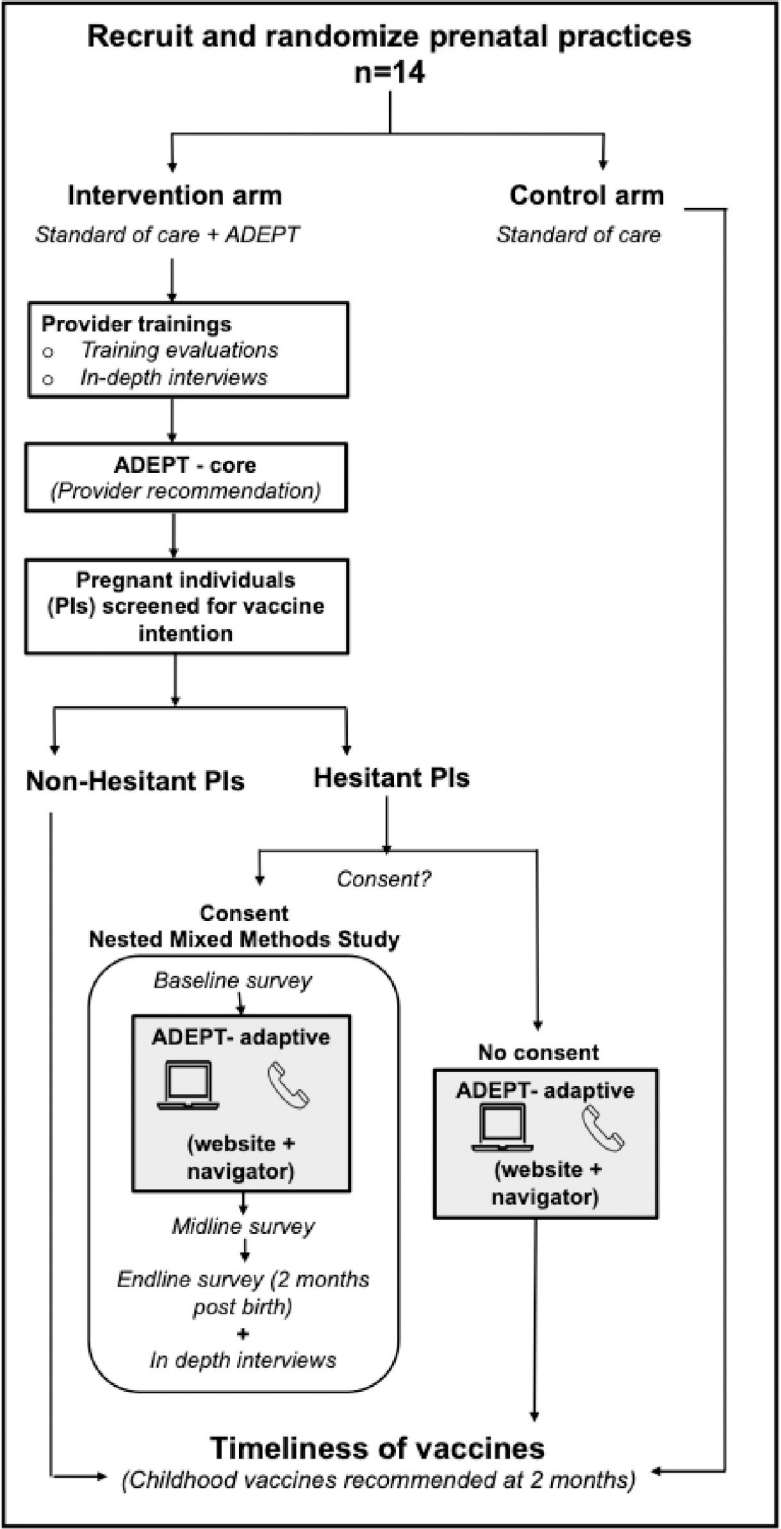
Practice and participant flow between the cluster randomized trial and nested mixed methods study.

We will cluster-randomize 14 study practices in a 1:1 ratio to the intervention or control arm utilizing a covariate constrained randomization approach [23]. Practices in the intervention arm will implement the standard of care plus ADEPT whereas practices in the control arm will continue to implement the standard of care (no ADEPT, **Fig 3**). After the PIs in the intervention and control arms give birth, we will assess timeliness of the 2-month vaccines for the child using data from the state immunization registry. The primary study outcome will be the difference between the intervention and control arms in the timeliness of childhood vaccinations recommended for children at 2 months (see **Outcomes**).

#### Nested Mixed Methods (NMM)

The NMM study will follow an explanatory sequential design and include those PIs who are eligible to receive the adaptive intervention component(s) and who consent to research activities to provide feedback on the intervention. The quantitative component of the NMM study will consist of survey questionnaires, and the qualitative component will consist of in-depth interviews. The qualitative portion will occur after the quantitative data collection to help explain or elaborate on the quantitative findings. Specifically, the qualitative data will explore reasons for observed intervention effects and identify factors that can explain significant or non-significant findings. Using joint displays and narrative summaries, we will integrate qualitative and quantitative outcomes to explain any observed intervention effect [24].

### Study setting

The study will be conducted in prenatal practices affiliated with health departments and a large academic medical center located in North Carolina. Practices may include diverse prenatal providers including obstetricians, family medicine practitioners, midwives, nurse practitioners, and physician assistants. The expected annual birth cohort is approximately 5,800 babies at the academic medical center, irrespective of first-time parent status.

### Participants and eligibility criteria

The prenatal practices will be those based in North Carolina and who provide routine prenatal care to PIs. Childhood vaccination discussions with PIs should not be part of practice standard of care for eligibility.

Prenatal providers will include those working in intervention practices. There are no exclusions by provider type or training.

PIs will be first-time pregnant individuals who receive routine prenatal care in study practices. PIs who are at a high risk of preterm delivery will be excluded to ensure there is adequate time to deliver the adaptive intervention components. High risk pregnancy will include multiple pregnancy, known fetal congenital malformations or genetic abnormalities, or documented as at-risk by the prenatal provider. Due to the timing of the ADEPT core component, PIs will be in their late second trimester during intervention exposure. For the NMM, vaccine hesitant PIs who are at least 18 years of age, provide informed consent for enrolment in NMM, and plan to stay in the study area for at least 3 months after delivery, will be eligible.

### Recruitment, screening, and enrollment

#### Prenatal practices

Prenatal care practices will be recruited using existing networks of the study investigators and members of the study external advisory board. Recruitment information will include a link to an interest survey. Any practices that indicate an interest in study participation will be contacted by study staff. If eligible, additional information will be collected from the selected practices for covariate constrained cluster randomization. In addition, to facilitate intervention implementation, each prenatal care practice will be asked to nominate a site champion for participation in the overall Study Advisory Board (SAB). The SAB will meet with study staff during the active implementation period. During these virtual meetings, suggestions or concerns may be discussed including recruitment strategy, recruitment and retention statistics, implementation challenges and solutions, and other lessons learned. To resolve any practice-specific challenges, study staff will meet with the site champions in one-on-one meetings.

#### Prenatal providers

All prenatal providers and staff at prenatal practices randomized to the intervention arm will be eligible to participate in the provider training. For those individuals who provide informed consent, we will collect information on their characteristics (e.g., demographics, prior training, years in practice) and their feedback on training sessions. A subset of the providers will be invited to participate in in-depth interviews to discuss their experience making childhood vaccine recommendations to PIs.

#### PIs

We will seek a waiver of consent for the assessment of the study outcomes among PIs and their children in the CRT since outcome data will be extracted from the state immunization registry where they are routinely reported, and any identifying information will be managed by an honest broker who is independent from the study team. Vaccination outcomes will be assessed at the practice-level and compared between study arms. Recruitment for the NMM study will occur in the intervention arm among those who self-identify as vaccine hesitant per the screening questionnaire. PIs who are eligible for the NMM study will be contacted by study staff and only those who provide informed consent will be enrolled in the NMM study. Participation in the NMM study is voluntary; PIs who do not want to participate will still have access to the ADEPT adaptive components as part of the CRT. Trained study staff will conduct the informed consent process in person or over the phone, with an electronic signature documenting consent. For the qualitative portion of the NMM study, PIs will be selected for the interview at the time of the endline survey (2 months post birth) to include perspectives from those who vaccinated their children on time, as well as those who delayed or refused vaccines at 2 months, according to CDC’s Birth-18 years immunization schedule (**S4 File**).

### Data collection

#### Provider trainings

Quantitative and qualitative data will be collected as a part of the provider training. Post-training evaluation surveys will capture feedback on training sessions, including the format of training, and delivery. Evaluation surveys will be self-administered by the providers after each of the four training sessions. All providers who complete the trainings, regardless of participation in post-training evaluations, will be eligible for continuing medical education (CME) credits, including Maintenance of Certification (MOC) Part 4 credits from American Board of Obstetrics and Gynecology (ABOG) [25]. After the trainings have been completed, interviews will be conducted with a subset of 12 prenatal providers from intervention practices to assess any additional training needs and to better understand the experience of implementing vaccine recommendations in practice. These interviews may be completed in-person or virtually and will be conducted in English by a trained interviewer. Each interview is expected to last approximately 15 minutes and will be audio-recorded for transcription and analysis purposes.

#### CRT

Representatives from each practice will complete a survey to provide data on the practice-level covariates necessary for constrained randomization and to assess readiness for implementation of ADEPT. At the time of outcome assessment, study practices will utilize medical records to generate a list of first-time PIs who were in their second and third trimester during the study period. The linkage of these data to the state immunization registry will be used to determine vaccination outcomes for children post-delivery. Identifiable data will be managed by an honest broker who will communicate with the state immunization registry for receiving vaccination data. De-identified datasets will be shared with the study team by the honest broker for calculation of vaccination outcomes.

#### NMM

For the NMM study, each participant will complete the screening questionnaire to determine study eligibility, followed by a baseline survey, a midline survey (prior to birth of child), and an endline survey. Survey domains will include intent to vaccinate self and child, vaccine confidence (Emory Vaccine Confidence Index [26]), health information seeking patterns, and demographics. Midline and endline surveys will evaluate acceptability of the adaptive components of the intervention. Prior to the surveys, each participant will complete an informed consent form, which will describe study processes, risks and benefits. Qualitative data will be collected using open-ended questions and prompts in individual in-depth interviews from participants who completed the endline surveys. Interview question domains may include reasons for delayed or timely vaccinations, feedback on intervention components, and residual information needs. Interviews will be held in English, over the phone or in person and are expected to last approximately 30 minutes each. The interview guide themes and questions will be influenced by the quantitative outcomes and used to benefit from further exploration. Interviews will be audio recorded to facilitate data transcription and analysis.

### Statistical considerations

#### Sample sizes

*Provider trainings*. Only individuals from the intervention practices will be offered access to the trainings. All providers, staff and trainees at these practices will be eligible to participate in the training.

*CRT*. We anticipate the proportion of children who receive delayed or no vaccination at 2 months of age at baseline to be 20% [6]. We estimate that the intervention will reduce this proportion to 10%. We used a sample size formula for a two-sided Z-test comparing two proportions that accounts for the CRT design and incorporates a small-sample bias correction suitable for CRTs with fewer than 30–40 clusters in total (see equation (3) of Thomson et al, and more details in **S5 File**) [27]. With a sample size of 7 practices in each arm (14 total) and with at least 55 PIs/practice (770 total) the study would be powered at 90% to detect a difference in the proportion of 10% between arms at a two-sided alpha level of 5%, assuming that the coefficient of variation of cluster (i.e., practice-level) proportions with the primary outcome is 0.15 [27]. The target sample size is further inflated by 10% (total: 850) to accommodate a small reduction in power anticipated due to variable enrollment by practice and a small proportion of children with missing effectiveness outcomes due to out-of-state movement [28, 29]. In reality, the sample size for each practice will depend on practice size and eligibility of the PI population seen during the study period. Given that the primary outcome will be measured via data linkage of practice-level data to the state immunization registry using an honest broker, we will request an extract of data for all eligible PIs who are seen at the practice during the study period with the goal that the majority of enrolled practices would be expected to meet this target. Sensitivity analyses show that 80% power would be achieved if the proportion of children who delayed or refused vaccination at 2 months of age is 30% (rather than 20%) with a slightly smaller CV of 0.125 or even if the coefficient of variation is as large as 0.2 (**S5 File**).

*NMM*. Sample size for the quantitative surveys will be estimated with the assumption that 20% of 425 PIs in the intervention arm are vaccine hesitant, and, hence, 85 PIs will be eligible to receive the adaptive intervention components as part of the NMM study. Of these, 68 are expected to agree to participate and to have midline survey data available (accounting for loss to follow-up) and estimate that the intervention will reduce the proportion of vaccine-hesitant to 80% at that time point. Using exact binomial methods, data from 68 PIs in a non-clustered setting would provide a 95% confidence interval ranging from 68% to 89% for a proportion vaccine-hesitant of 80% with a small increase due to the small design effect anticipated due to clustering. For the qualitative portion, existing research suggests that thematic saturation can be achieved with 12 interviews, with meta-themes presenting as early as six interviews [30, 31]. The proposed sample size of 24 PIs should be sufficient to achieve thematic saturation of reasons for delayed/refused vaccinations (n = 12) or timely vaccinations (n = 12).

### Randomization

An independent biostatistician at the study institution will generate the randomization sequence. Practices will be allocated using a 1:1 ratio to the intervention or control arm. A covariate constrained randomized approach will be implemented in order to facilitate comparability between arms in practice-level characteristics [32]. Practices will first be stratified by type (e.g., based on their affiliation with the academic medical center or the health department). Within strata, the following covariates will be considered: Practice size (quantified as # providers or patient volume), Race/ethnicity of the PI (African American vs not, Latinx vs. not i.e. two variables), and Insurance type of the PI. The choice of the race/ethnicity variable is based on our prior findings suggesting higher hesitancy among self-identified Black or African American pregnant individuals [9]. Final covariate selection will depend on data quality and completeness. There is no randomization of participants in the NMM study.

### Outcomes

The primary study outcome is the proportion of children receiving timely vaccinations at 2 months post birth, by study arm. The secondary outcome is change in vaccine hesitancy among PIs who receive the adaptive components of the intervention, within the intervention arm only, where change is measured from baseline to midline of the NMM (i.e., before and after receiving ADEPT intervention).

### Data analysis

All analyses of quantitative outcomes will utilize analytic software, such as SAS or STATA, and qualitative analyses will be conducted utilizing software such as MaxQDA.

#### Provider training

Summary characteristics for consenting providers will be described by study practice. Summary statistics will be calculated from data on provider training. Broad thematic domains from interviews with providers will be identified and cross referenced with quantitative data.

#### CRT

Summary characteristics for study practices will be described by intervention and control arm. We will also describe the distribution of vaccination intention among PIs. A timeframe of 28 days from the vaccination due date will be used as the window of timeliness. Timely vaccination doses will receive a score of 2, doses that are delayed beyond the 28-day window will receive a score of 1, and the non-receipt of a dose during outcome assessment will be scored a 0. For the time point of outcome determination (2 months), we will create a cumulative timeliness score and use that to determine the proportion of children who receive all recommended vaccines in a timely fashion. For the analysis of the primary outcome of vaccination timeliness, we will use data on vaccination outcomes for children of all PIs enrolled in the study (including those who screened as vaccine-hesitant in the intervention arm). The generalized estimating equations (GEE) approach will be used to account for clustering of outcomes due to the hierarchical structure of data (PIs nested within practice). The child-level binary outcome (timely vs. not) will be regressed on an intervention-arm indicator, strata and the practice-level covariates used in the covariate constrained randomization procedure. We note that no additional adjustment by individual-level characteristics is possible due to the source of data, i.e., state immunization registry. Log and identity links will be used to obtain both relative and absolute intervention effects, as per recommendations from the CONSORT statement on trial reporting [22]. Robust standard errors will be used together with finite-sample corrections to reduce inflated Type I error rate, which is expected due to the small numbers of clusters (I.e. 14 practices) [33]. Given that outcomes are obtained from the state immunization registry, we do not expect missing data in the usual sense. Instead, some outcome data will not be available for those children whose birthing parents were enrolled towards the end of the study. Since this is by design, the missing data is not expected to bias the estimated intervention effect. A similar GEE approach will be used to model vaccination coverage.

#### NMM

Summary characteristics of PIs enrolled and receiving the adaptive components of the intervention will be computed. The secondary outcome of the study, change in vaccine hesitancy from baseline to midline, will be computed utilizing data collected from enrolled PIs prior to receipt of the intervention, and at midline (up to 2 months post intervention, prior to birth). Given the anticipated sample size of PIs enrolled in the NMM study, we anticipate being underpowered to detect any meaningful pre-post difference. However, the determination of this outcome is important to inform sample size estimates for future studies.

Thematic analysis of qualitative data will occur using a phenomenological lens using the following steps: A) an initial read through of the transcripts to evaluate content and quality, b) coding of a priori themes based on the interview guide during a second read through and an additional review of 10% sample coded transcripts by a secondary team member, c) display of data through patterns and subthemes/codes and descriptions in thematic memos, and D) data reduction by applying matrices to organize and extract the key concepts on vaccine hesitancy.

Mixed methods data integration will occur using a joint display. Qualitative themes explaining the vaccination decision and describing residual barriers will be organized by a joint display by quantitative outcomes as follows: ADEPT and timely vaccinations, ADEPT, and delayed vaccinations. The joint display will be used to facilitate an explanation of the impact of ADEPT on timely vaccination [24].

### Study strengths and limitations

There may be lower rates of vaccine-hesitant PI enrollment in the NMM study because of the reliance of self-selection. We plan to employ diverse recruitment methods (including patient portal messages, flyers, etc.) to help mitigate this risk. If lower enrollment is identified during tracking, higher usage of purposive recruitment methods may be used. In the unlikely event that reasonable balance in study practice characteristics is not achieved using the constrained randomization technique, the team will explore other statistical approaches to minimize confounding effects like adjusting for imbalanced covariates in statistical models. Despite exclusions of high-risk PIs, there is always the possibility of early delivery for participants (before 36 weeks) or patient drop-off, which leaves less time for implementation of the adaptive intervention components. However, clinician experts will evaluate the inclusion/exclusion criteria, and the research team will tailor analysis if such concerns arise. Lastly, due to the varying size of the practices, there may be variable recruitment rates and numbers. However, there is no reason to expect differential dropout rates between study arms. These potential problems are also accounted for in our sample size estimates by incorporating a 10% inflation rate.

### Ethical approval and considerations

The study protocol was approved by the Duke University Health System Institutional Review Board (Pro00109337, Pro00110413, Pro00110099) and Emory University’s Institutional Review Board (Authorization agreement for reliance on DUHS IRB; 00005457, 00005432, 00005431). The Centers for Disease Control and Prevention (CDC) only has access to de-identified data and therefore determined that the CDC was not engaged in human subjects research and CDC’s IRB approval was not required. The study was prospectively registered on ClinicalTrials.gov (ID: NCT05795855).

## Conclusions

The evaluation of ADEPT seeks to bridge gaps in the evidence on the effectiveness of discussing childhood vaccinations proactively during a routine prenatal care visit. The ADEPT study is expected to contribute training materials on childhood vaccinations for prenatal providers, data on ADEPT’s effectiveness in promoting childhood vaccinations compared to standard of care and inform future implementation and evaluation of ADEPT in diverse prenatal settings across the US. These contributions are significant because they will advance our knowledge of strategies for mitigating vaccine hesitancy, which is a substantial barrier to achieving the Healthy People 2030 goals to reduce the proportion of un- or under-immunized children in the United States.

## Supporting information

S1 File SPIRIT 2013 checklist: Recommended items to address in a clinical trial protocol and related documents. Citation: Chan A-W, Tetzlaff JM, Altman DG, Laupacis A, Gøtzsche PC, Krleža-Jerić K, Hróbjartsson A, Mann H, Dickersin K, Berlin J, Doré C, Parulekar W, Summerskill W, Groves T, Schulz K, Sox H, Rockhold FW, Rennie D, Moher D. SPIRIT 2013 Statement: Defining standard protocol items for clinical trials. Ann Intern Med. 2013;158(3):200–207.
(DOC)

S2 File The TIDieR (Template for Intervention Description and Replication) checklist. Citation: Hoffmann T, Glasziou P, Boutron I, Milne R, Perera R, Moher D, Altman D, Barbour V, Macdonald H, Johnston M, Lamb S, Dixon-Woods M, McCulloch P, Wyatt J, Chan A, Michie S. Better reporting of interventions: template for intervention description and replication (TIDieR) checklist and guide. BMJ. 2014;348:g1687.
(DOCX)

S3 File CONSORT 2010 checklist of information to include when reporting a cluster randomised trial. Citation: Campbell MK, Piaggio G, Elbourne DR, Altman DG. Consort 2010 statement: extension to cluster randomised trials. BMJ: British Medical Journal. 2012;345:e5661.
(DOCX)

S4 File Recommended childhood immunizations at 2 months, United States, 2024. Citation: U. S. Centers for Disease Control and Prevention. Recommended Child and Adolescent Immunization Schedule for Ages 18 Years or Younger, United States, 2024. Accessed September 17, 2024.
(DOCX)

S5 File Details of the sample size calculations for the ADEPT study cluster randomized trial. (DOCX)
