# Supplementary material for: An ADaptivE PrenaTal (ADEPT) intervention to increase childhood vaccinations: Protocol for a cluster randomized trial and nested mixed methods evaluation
Source: PLoS One. 2024 Nov 21;19(11):e0313742. doi: 10.1371/journal.pone.0313742 (PMC11581330; doi:10.1371/journal.pone.0313742)
Supplement: S4 File — Citation: U. S. Centers for Disease Control and Prevention. Recommended Child and Adolescent Immunization Schedule for Ages 18 Years or Younger, United States, 2024. Accessed September 17, 2024. (DOCX) [file pone.0313742.s004.docx]

**S4 File: Recommended Childhood Immunizations at 2 months, United States, 2024**

| **Product** | **Dose** | **Notes** |
| --- | --- | --- |
| Respiratory syncytial virus  (RSV-mAb [Nirsevimab]) | 1^st^ | Recommended if not already received before 2 months of age AND if birthing parent did not receive vaccine during pregnancy |
| Hepatitis B (HepB) | 2^nd^ | Recommended if not already received at 1 month of age |
| Rotavirus (RV): RV1 (2-dose series), RV5 (3-dose series) | 1^st^ |  |
| Diphtheria, tetanus, acellular pertussis (DTaP <7 yrs) | 1^st^ |  |
| *Haemophilus influenzae* type b (Hib) | 1^st^ |  |
| Pneumococcal conjugate (PCV15, PCV20) | 1^st^ |  |
| Inactivated poliovirus (IPV <18 yrs) | 1^st^ |  |

**Source:** U. S. Centers for Disease Control and Prevention. Recommended Child and Adolescent Immunization Schedule for Ages 18 Years or Younger, United States, 2024. Accessed September 17, 2024.
