# Supplementary material for: An ADaptivE PrenaTal (ADEPT) intervention to increase childhood vaccinations: Protocol for a cluster randomized trial and nested mixed methods evaluation
Source: PLoS One. 2024 Nov 21;19(11):e0313742. doi: 10.1371/journal.pone.0313742 (PMC11581330; doi:10.1371/journal.pone.0313742)
Supplement: S5 File — (DOCX) [file pone.0313742.s005.docx]

**S5 File: Details of the sample size calculations for the ADEPT study cluster randomized trial.**

Using equation (3) of Thomson et al., we estimated the number of clusters needed per arm (c) to detect a 10-percentage point difference between arms assuming data from 55 mother-child pairs for each practice. That equation is reproduced here:

$$\begin{matrix} c=1+(Z_{\alpha/2}+Z_{\beta})^{2}\frac{\left[\frac{\Pi_{0}\left(1-\Pi_{0}\right)+\Pi_{1}\left(1-\Pi_{1}\right)}{n}+k^{2}\left(\Pi_{0}^{2}+\Pi_{1}^{2}\right)\right]}{\left(\Pi_{0}-\Pi_{1}\right)^{2}} \\ \end{matrix}$$

We assumed the following parameter values for the primary outcome of the proportion of children who receive delayed or no vaccination at 2 months of age, which led to an estimated sample size of 7 clusters per arm with primary outcome data from 55 mother-child dyads.

| **Component** | **Symbol** | **Value for**  **vaccination study** |
| --- | --- | --- |
| Number of subjects per cluster | n | 55 |
| Coefficient of variation | k | 0.15 |
| Power | 1-β | 0.90 |
| Z for power | Z_β_ | -1.28 |
| Alpha | α | 0.05 |
| Z for alpha-level | Z_α/2_ | -1.96 |
| Proportion with primary outcome in control arm | Π_0_ | 0.20 |
| Proportion with primary outcome in intervention (ADEPT) arm | Π_1_ | 0.10 |

We note that the formula above accounts for small-sample bias by the addition of 1 cluster per arm to account for the fact that, otherwise, a Z-based formula may not be appropriate. An alternative strategy would be to use a t-based formula but given that the percentiles would need to change based on the number of clusters per arm (*c*), we instead adopted the validated approach of Thomson et al. (2009).

Furthermore, the coefficient of variation (CV) was calculated using an approach outlined by Thomson et al. (2009). Specifically, we assumed that the control-arm cluster-specific outcome proportions were centered on the value of 20% and that 95% of them lie between 14% and 26% in which case, the half-width of that interval is 6%, corresponding to a standard deviation of the control cluster-specific proportions of 3%. Therefore, the CV is estimated to be 3% / 20% = 0.15.

Reference: Thomson A, Hayes R, Cousens S. Measures of between-cluster variability in cluster randomized trials with binary outcomes. Stat Med. 2009 May 30;28(12):1739-51. doi: 10.1002/sim.3582. PMID: 19378266.

| **Sample**  **Size per Cluster** | **Control**  **Proportion** | **Intervention**  **Proportion** | **Coefficient of Variation** | **80% Power** | | **85% Power** | | **90% Power** | |
| --- | --- | --- | --- | --- | --- | --- | --- | --- | --- |
|  |  |  |  | **Number of Clusters per arm** | **Sample**  **Size**  **per arm** | **Number of Clusters per arm** | **Sample**  **Size**  **per arm** | **Number of Clusters per arm** | **Sample**  **Size**  **per arm** |
| 40 | 0.2 | 0.1 | 0.125 | 7 | 280 | 8 | 320 | 9 | 360 |
|  |  |  | 0.150 | 7 | 280 | 8 | 320 | 9 | 360 |
|  |  |  | 0.200 | 8 | 320 | 9 | 360 | 10 | 400 |
| 40 | 0.3 | 0.2 | 0.125 | 10 | 400 | 12 | 480 | 13 | 520 |
|  |  |  | 0.150 | 11 | 440 | 12 | 480 | 14 | 560 |
|  |  |  | 0.200 | 13 | 520 | 14 | 560 | 17 | 680 |
| 45 | 0.2 | 0.1 | 0.125 | 6 | 270 | 7 | 315 | 8 | 360 |
|  |  |  | 0.150 | 7 | 315 | 7 | 315 | 9 | 405 |
|  |  |  | 0.200 | 7 | 315 | 8 | 360 | 9 | 405 |
| 45 | 0.3 | 0.2 | 0.125 | 10 | 450 | 11 | 495 | 12 | 540 |
|  |  |  | 0.150 | 10 | 450 | 11 | 495 | 13 | 585 |
|  |  |  | 0.200 | 12 | 540 | 14 | 630 | 16 | 720 |
| 50 | 0.2 | 0.1 | 0.125 | 6 | 300 | 7 | 350 | 8 | 400 |
|  |  |  | 0.150 | 6 | 300 | 7 | 350 | 8 | 400 |
|  |  |  | 0.200 | 7 | 350 | 8 | 400 | 9 | 450 |
| 50 | 0.3 | 0.2 | 0.125 | 9 | 450 | 10 | 500 | 11 | 550 |
|  |  |  | 0.150 | 10 | 500 | 11 | 550 | 12 | 600 |
|  |  |  | 0.200 | 11 | 550 | 13 | 650 | 15 | 750 |
| 55 | 0.2 | 0.1 | 0.125 | 6 | 330 | 6 | 330 | 7 | 385 |
|  |  |  | 0.150 | 6 | 330 | 7 | 385 | 7 | 385 |
|  |  |  | 0.200 | 7 | 385 | 7 | 385 | 8 | 440 |
| 55 | 0.3 | 0.2 | 0.125 | 8 | 440 | 9 | 495 | 11 | 605 |
|  |  |  | 0.150 | 9 | 495 | 10 | 550 | 12 | 660 |
|  |  |  | 0.200 | 11 | 605 | 12 | 660 | 14 | 770 |
| 60 | 0.2 | 0.1 | 0.125 | 5 | 300 | 6 | 360 | 7 | 420 |
|  |  |  | 0.150 | 6 | 360 | 6 | 360 | 7 | 420 |
|  |  |  | 0.200 | 6 | 360 | 7 | 420 | 8 | 480 |
| 60 | 0.3 | 0.2 | 0.125 | 8 | 480 | 9 | 540 | 10 | 600 |
|  |  |  | 0.150 | 9 | 540 | 10 | 600 | 11 | 660 |
|  |  |  | 0.200 | 10 | 600 | 12 | 720 | 13 | 780 |
| *Shaded areas indicate samples sizes that provide at least 80% power.* | | | | | | | | | |

**Supplemental information regarding sample size for primary outcome for the CRT**
